# Extracellular Vesicles From Hepatocytes Are Therapeutic for Toxin-Mediated Fibrosis and Gene Expression in the Liver

**DOI:** 10.3389/fcell.2019.00368

**Published:** 2020-01-10

**Authors:** Xinlei Li, Ruju Chen, Sherri Kemper, David R. Brigstock

**Affiliations:** ^1^Center for Clinical and Translational Research, The Research Institute at Nationwide Children’s Hospital, Columbus, OH, United States; ^2^Department of Surgery, Wexner Medical Center, The Ohio State University, Columbus, OH, United States

**Keywords:** extracellular vesicle, hepatocyte, hepatic stellate cell, hepatic fibrosis, inflammation, cell cycle, extracellular matrix, exosome

## Abstract

Extracellular vesicles (EVs) are nano-sized membrane-limited organelles that are liberated from their producer cells, traverse the intercellular space, and may interact with other cells resulting in the uptake of the EV molecular payload by the recipient cells which may become functionally reprogramed as a result. Previous *in vitro* studies showed that EVs purified from normal mouse AML12 hepatocytes (“EV^Norm^”) attenuate the pro-fibrogenic activities of activated hepatic stellate cells (HSCs), a principal fibrosis-producing cell type in the liver. In a 10-day CCl_4_ injury model, liver fibrogenesis, expression of hepatic cellular communication network factor 2 [CCN2, also known as connective tissue growth factor (CTGF)] or alpha smooth muscle actin (αSMA) was dose-dependently blocked during concurrent administration of EV^Norm^. Hepatic inflammation and expression of inflammatory cytokines were also reduced by EV^Norm^. In a 5-week CCl_4_ fibrosis model in mice, interstitial collagen deposition and mRNA and/or protein for collagen 1a1, αSMA or CCN2 were suppressed following administration of EV^Norm^ over the last 2 weeks. RNA sequencing (RNA-seq) revealed that EV^Norm^ therapy of mice receiving CCl_4_ for 5 weeks resulted in significant differences [false discovery rate (FDR) <0.05] in expression of 233 CCl_4_-regulated hepatic genes and these were principally associated with fibrosis, cell cycle, cell division, signal transduction, extracellular matrix (ECM), heat shock, cytochromes, drug detoxification, adaptive immunity, and membrane trafficking. Selected gene candidates from these groups were verified by qRT-PCR as targets of EV^Norm^ in CCl_4_-injured livers. Additionally, EV^Norm^ administration resulted in reduced activation of p53, a predicted upstream regulator of 40% of the genes for which expression was altered by EV^Norm^ following CCl_4_ liver injury. *In vitro*, EVs from human HepG2 hepatocytes suppressed fibrogenic gene expression in activated mouse HSC and reversed the reduced viability or proliferation of HepG2 cells or AML12 cells exposed to CCl_4_. Similarly, EVs produced by primary human hepatocytes (PHH) protected PHH or human LX2 HSC from CCl_4_-mediated changes in cell number or gene expression *in vitro*. These findings show that EVs from human or mouse hepatocytes regulate toxin-associated gene expression leading to therapeutic outcomes including suppression of fibrogenesis, hepatocyte damage, and/or inflammation.

## Introduction

A common pathophysiological feature of chronic liver injury is the excessive production of collagen and extracellular matrix (ECM) components that become deposited as insoluble fibrotic scar material, resulting in physical and biochemical changes which can compromise essential cellular functions. Hepatic stellate cells (HSC) are the major fibrosis-producing cell type in the liver and their fibrogenic actions result from their participation in an orchestrated molecular response to sustained injury that also involves injured hepatocytes, activated resident macrophages, sinusoidal endothelial cells, and infiltrating immune cells. Many studies have documented that numerous soluble or ECM-associated signaling molecules comprise a complex regulatory network that drives and fine-tunes the fibrogenic response via autocrine, paracrine, or matricellular pathways among these various cell types. HSC function is also regulated by juxtacrine pathways that are triggered by direct cell-cell contact. In turn, these studies have highlighted the potential value of targeting components of these pathways in the development of new-generation anti-fibrotics (Ghiassi-Nejad and Friedman, 2008; Cohen-Naftaly and Friedman, 2011). However, in many cases the optimal targets, type of therapeutic agent and mode of administration have yet to be elucidated and there remain few viable options for direct anti-fibrotic therapy in patients whose liver fibrosis does not resolve upon treatment of their primary liver disease.

A newly recognized component of cell-cell communication in the liver is intercellular trafficking of molecules that are contained within nano-sized extracellular vesicles (EVs) such as exosomes or microvesicles. Whereas microvesicles are generally 200–1000 nm in diameter and are formed by budding of the plasma membrane, exosomes are typically 50–200 nm in diameter and arise by inward budding of multivesicular bodies which then fuse with the plasma membrane and cause exosomes to be released from the cell. Despite their distinct pathways of biogenesis, both exosomes and microvesicles mediate cell-cell transfer of their respective molecular payloads that can potentially cause reprograming events in recipient cells. EV cargo molecules include a broad spectrum of microRNAs, mRNAs and proteins that originate from their cells of origin and which are protected from extracellular degradation by the presence of the outer vesicular membrane which also functions to present docking proteins and receptors to recipient cells, allowing for EV uptake either by fusion with the plasma membrane or by endocytosis (Kowal et al., 2016). A role for liver-derived exosomes in fibrosis is suggested by an increase in activation or fibrogenesis in HSC after their exposure to exosomes from lipid-laden hepatocytes (Povero et al., 2015), sinusoidal endothelial cells (Wang et al., 2015), or from activated HSC themselves, the latter of which involved EV-mediated delivery of pro-fibrogenic cellular communication network factor 2 (CCN2), also known as connective tissue growth factor (CTGF; Charrier et al., 2014a; Perbal et al., 2018). However, the molecular composition of the EV molecular payload and the phenotypic status of the recipient cells are critical determinants of how EV action is manifested in the context of liver fibrosis. For example, we have shown that quiescent HSC produce exosomes that attenuate pathways of activation or fibrogenesis in activated HSC due to exosomal transfer into the recipient cells of miR-214 or miR-199a-5p which directly inhibit transcription of CCN2, thus suppressing downstream alpha smooth muscle actin (αSMA) or collagen production and reverting the cells to a more quiescent phenotype (Chen et al., 2014, 2015, 2016b). Hence the molecular cargo in exosomes from HSC may be pro- or anti-fibrogenic depending on the differential activation status of producer versus recipient cell. Similarly, EVs produced by fat-laden hepatocytes drive pro-fibrogenic pathways in HSC (Povero et al., 2015) whereas we recently showed that EVs from normal hepatocytes bind to HSC *in vivo* and have suppressive actions on activated HSC *in vitro* (Chen et al., 2018a). In pursuing the latter findings, we now report that EVs from hepatocytes have anti-fibrotic actions *in vivo* that are associated with attenuated HSC activation and fibrogenesis, hepatocyte recovery, reduced levels of hepatic monocytes and macrophages, and attenuated expression of inflammatory mediators, ECM components, detoxifying cytochromes, and regulators of cell division. These findings reveal that EVs from hepatocytes have previously unrecognized therapeutic actions in the liver.

## Materials and Methods

### CCl_4_-Induced Hepatic Fibrogenesis in Mice

Animal protocols were approved by the Institutional Animal Care and Use Committee of Nationwide Children’s Hospital (Columbus, OH, United States). Male Swiss Webster wild type mice or transgenic (TG) Swiss Webster mice expressing enhanced green fluorescent protein (EGFP) under the control of the promoter for CCN2 (TG CCN2-EGFP (Charrier et al., 2014b); (4–5 weeks old; *n* = 5 per group) were injected i.p. with carbon tetrachloride (CCl_4_; 4 μl in 26 μl corn oil/25 g body weight; Sigma-Aldrich, St. Louis, MO, United States) on Days 1, 3, 5, and 8. Control mice received i.p. corn oil (30 μl/25 g) on the same days. Some mice received i.p. mouse hepatocyte EVs (prepared as described below; 0–80 μg EV protein per 25 g body weight) on Days 2, 4, 6, and 9. Mice were sacrificed 2 days after the last injection and liver lobes were either perfused with phosphate-buffered saline (PBS), fixed in 4% paraformaldehyde and processed for histological analysis or immediately harvested for EGFP imaging using a Xenogen IVIS 200 (PerkinElmer, Waltham, MA, United States) or snap-frozen in liquid nitrogen for later RNA or protein extraction.

### CCl_4_-Induced Hepatic Fibrosis in Mice

Wild-type male Swiss Webster mice (4–5 weeks old; *n* = 5 per group) were injected i.p. with CCl_4_ (4 μl in 26 μl corn oil/25 g) or corn oil (30 μl/25 g) three times per week for 5 weeks. On alternative days to those used for CCl_4_ or oil administration, some mice received i.p. EV (0–80 μg/25 g) three times per week over the last 2 weeks of the experiment. Mice were sacrificed 36 h after the last CCl_4_ or oil injection, or in non-treated littermates. Individual liver lobes were harvested and snap-frozen in liquid nitrogen for subsequent RNA extraction or perfused using PBS followed by 4% paraformaldehyde (Sigma-Aldrich) for histological analysis of fixed tissue.

### Histology

Perfused mouse livers were fixed and embedded in paraffin. Sections of 5 μm thickness were cut and stained with H and E. Sections were stained with 0.1% Sirius Red (Sigma-Aldrich) for detection of collagen or subjected to immunohistochemistry (IHC) (see below). Positive signals were quantified by image analysis.

### Hepatocyte Cultures

AML12 mouse hepatocytes [American Type Culture Collection (ATCC), Manassas, VA, United States] were maintained *in vitro* in DMEM/F12/10% FBS containing insulin, transferrin, selenium and dexamethasone (Chen et al., 2015). HepG2 cells (ATCC) were maintained in DMEM/10% FBS. Primary human hepatocytes (PHH; IVAL LLC, Columbia, MA, United States) were cultured in Universal Primary Cell Plating Medium (IVAL) according to the vendors’ directions.

### Hepatic Stellate Cell Cultures

Primary mouse HSC were isolated from male wild-type Swiss Webster mice (6–8 week) by perfusion and digestion of livers with pronase/collagenase, followed by buoyant-density centrifugation (Ghosh and Sil, 2006; Chen et al., 2011, 2016b). The cells were maintained in Gibco DMEM/F12/10% FBS medium (Thermo Fisher Scientific, Waltham, MA, United States) and split every 5 days for use up to passage 4 (P4). Freshly isolated quiescent HSC contained lipid droplets that we previously showed to express desmin or Twist1 but not CCN2, αSMA or collagen 1a1 (Col1a1) whereas cells that autonomously activated over the first week of culture expressed desmin, CCN2, αSMA or Col1a1, but not Twist1 (Chen et al., 2011, 2015). LX-2 human HSC (courtesy of Dr. Scott Friedman, Icahn School of Medicine at Mount Sinai, New York, NY, United States) were cultured in DMEM/10% FBS as described (Chen et al., 2011, 2015).

### Hepatocyte EV Purification

Prior to collecting EVs from the conditioned medium of mouse or human hepatocytes, spent medium was removed, and the cells were rinsed twice with Hanks Balanced Salt Solution prior to incubation overnight in their respective serum-free medium which was then replaced with fresh serum-free medium for up to 48 h. The supernatant obtained from sequential centrifugation of conditioned medium (300 × *g* for 15 min, 2,000 × *g* for 20 min, 10,000 × *g* for 30 min) underwent ultracentrifugation (100,000 × *g* for 70 min at 4°C) in a T-70i fixed-angle rotor (Beckman Coulter, Brea, CA, United States), the pellet from which was resuspended in PBS and subjected to the same ultracentrifugation conditions again. The resulting pellet was dispersed in PBS and the constituent EVs were characterized as described below. AML12 cell-derived EVs are hereafter termed “EV^Norm^” since they were produced under essentially normal culture conditions as opposed to other EV studies involving hepatocyte injury or stress (Povero et al., 2013, 2015; Momen-Heravi et al., 2015; Hirsova et al., 2016a; Ibrahim et al., 2016; Verma et al., 2016; Lee et al., 2017).

### Nanoparticle Tracking Analysis (NTA)

EVs in PBS (10^7^–10^8^ particles/ml) were subjected to nanoparticle tracking analysis (NTA) using a Nanosight 300 (Malvern Instruments, Westborough, MA, United States) that had been calibrated with 100 nm polystyrene latex microspheres (Magsphere Inc., Pasadena, CA, United States) diluted in PBS to a fixed concentration. EV samples were each analyzed twice and v3.2.16 analytical software (Malvern Instruments) was used to estimate mean particle concentration and size. Recordings were performed at room temperature with a camera gain of 15 and a shutter speed of 4.13 ms. The detection threshold was set to 6.

### Transmission Electron Microscopy

EV^Norm^ were fixed in 2.5% glutaraldehyde for 30 min and pelleted by ultracentrifugation. They were then placed on 200-mesh copper grids that had been coated with Formvar/Carbon support film (Ted Pella, Redding, CA, United States) and glow-discharged with a PELCO easiGlow discharge cleaning system (Ted Pella). EVs were negatively stained with 1% aqueous uranyl acetate and examined by transmission electron microscopy (TEM) using a FEI Tecnai G2 Biotwin instrument (Atlanta, GA, United States) operating at 80 kV. Digital micrographs were captured using an AMT camera with FEI imaging software.

### EV Cell Binding Assays

Primary human hepatocytes-derived EVs were labeled with the fluorescent lipophilic membrane dye PKH26 (Sigma-Aldrich) essentially as previously described (Chen et al., 2018a) and incubated in a 96-well culture plate at 2 × 10^9^ particles/ml for 24 h with PHHs (on collagen-coated wells) or LX-2 cells that had been exposed to 20 mM CCl_4_ for the preceding 24 h. Cells were stained with DAPI, rinsed in PBS, and EV binding was assessed using a LSM 800 fluorescence microscope (Carl Zeiss Inc., Thornwood, NY, United States) and quantified using ImageJ software (NIH, Bethesda, MD, United States).

### Effect of Hepatocyte EVs on Hepatocyte or HSC Function *in vitro*

HepG2 cells, PHHs, or human LX2 HSC were incubated for 24 h in their respective normal growth media prior to being placed under fresh serum-free conditions in the presence or absence of 20 mM CCl_4_ for 48 h, the last 24 h of which included incubation with or without EVs from HepG2 cells or PHHs (8–16 μg/ml or 2–4 × 10^8^ particles/ml, respectively). Cells were assessed for cell proliferation using a CyQUANT^®^ assay (Thermo Fisher Scientific) or for viability using an AlamarBlue assay (Thermo Fisher Scientific). For gene expression studies, PHHs, LX2 cells, or mouse P3-4 HSC were placed in fresh serum-free medium overnight prior to treatment with EVs from HepG2 cells (8 μg/ml) or PHHs (4 × 10^8^ particles/ml) for 36–48 h in the presence or absence of 20 mM CCl_4_ prior to analysis by RT-PCR.

### Iodixanol Gradient Ultracentrifugation

PKH26-labeled EVs in 200 μl PBS were loaded on to a 5 ml stepwise gradient of 8, 16, 24, 32, and 40% iodixanol followed by ultracentrifugation at 37,500 rpm (SW55 Ti rotor, Beckman) for 17 h at 4°C. The tube contents were collected from top to bottom into 20 fractions which were assessed for iodixanol concentration using an Abbe refractometer (Bausch and Lomb, Rochester, NY, United States) and PKH26 fluorescence (Ex/Em = 540/585 nm, cut-off = 570 nm) using a Spectra Max M2 spectrophotometer (VWR, Atlanta, GA, United States). The fraction containing the peak PKH26 signal was then assessed for its ability to modulate gene expression in activated mouse HSC.

### Protein Assay

A Pierce bicinchoninic acid assay kit (Thermo Fisher Scientific) was used to quantify protein concentrations of EVs in PBS or of liver tissue proteins that had been extracted using Pierce radioimmunoprecipitation buffer (Thermo Fisher Scientific).

### Immunohistochemistry

Mouse liver sections were incubated with primary antibodies to F4/80 (1:100, Abcam, Cambridge, MA, United States), Ly6c (1:400, Abcam), collagen 1 (1:250, Abcam), αSMA (1:500; Thermo Fisher Scientific), or CCN2 [in-house; 5 μg/ml (Charrier and Brigstock, 2010; Charrier et al., 2014b)] followed by Alexa Fluor 488 goat-anti rabbit IgG and Alexa Fluor 568 goat-anti mouse IgG, or Alexa Fluor 647 goat-anti mouse IgG, or Alexa Fluor 568 goat-anti-chicken IgG (all at 1:500; Thermo Fisher Scientific) for 1 h at room temperature. The slides were mounted with Vectashield Mounting Medium containing 4′,6-diamidino-2-phenylindole nuclear stain (Vector Laboratories, Burlingame, CA, United States), and examined by confocal microscopy. Activated HSC were identified by positive immunostaining for CCN2, αSMA and/or Col1a1.

### Mouse Cytokine Microarray

A mouse cytokine proteome profiler^TM^ array (Panel A kit; R & D Systems, Minneapolis, MN, United States) was incubated with detergent lysates (320 μg/ml) of pooled liver tissue from acute CCl_4_ injury in male Swiss Webster mice (short-term fibrogenesis model) with or without EV^Norm^ treatment, as described above (*n* = 3 or 4 mice per group). Arrays were developed using chemiluminescence and the mean pixel intensity of duplicate component spots was quantified using ImageJ (NIH, Bethesda, MD, United States). Targets showing significant changes in intensity in response to CCl_4_ and EV^Norm^ were tested for their corresponding RNA expression by quantitative real-time polymerase chain reaction (qRT-PCR).

### Western Blot

Proteins in liver tissue extracts or hepatocyte EVs were subjected to sodium dodecyl polyacrylamide gel electrophoresis (SDS-PAGE; 10–25 μg protein/lane). Depending on the sample in question, Western blots were performed to detect proteins associated with EVs, cytosol, or liver fibrosis. Blots were incubated with primary antibodies to CD9 (1:500; Abcam), Alix (1:500; Thermo Fisher Scientific), TSG101 (1:500; Thermo Fisher Scientific), flotillin-1 (1:200; BD Biosciences, San Jose, CA, United States), CD63 (1:100; Millipore Sigma, Burlington, MA, United States), calnexin (1:500; Novus Biologicals, Centennial, CO, United States), calreticulin (1:500; Novus Biologicals, United States), p53 (1:1,000; Cell Signaling, Danvers, MA, United States), phopho-p53 at Ser15 (1:1,000; Cell Signaling) or β-actin (1:1,000; Invitrogen, Waltham, MA, United States). Blots were developed using an Odyssey Imaging System (LI-COR Biosciences, Lincoln, NE) and signals were normalized relative to β-actin for quantification using ImageJ (NIH).

### RNA Sequencing

RNA-seq libraries from mouse livers (long-term fibrosis model with *n* = 3 per group for control, oil, oil + EV^Norm^, CCl_4_, or CCl_4_ + EV^Norm^) were prepared using the TruSeq Stranded protocol (Illumina, San Diego, CA, United States). Following assessment of the quality of total RNA using an Agilent 2100 Bioanalyzer and RNA Nano chip (Agilent Technologies, Santa Clara, CA, United States), 100 ng mouse liver total RNA was treated with a Ribo-zero Human/Mouse/Rat Gold kit (Illumina) to deplete the levels of cytoplasmic and mitochondrial ribosomal RNA (rRNA) using biotinylated, target-specific oligos combined with rRNA removal beads. Following rRNA removal, mRNA was fragmented using divalent cations under elevated temperature and converted into double stranded cDNA. Incorporation of dUTP in place of dTTP during second strand synthesis inhibited the amplification of the second strand. The subsequent addition of a single “A” base allowed for ligation of unique, dual-indexed adapters with unique molecular identifiers (UMIs) (Integrated DNA Technologies, Coralville, IA, United States). Adaptor-ligated cDNA was amplified by limit-cycle PCR. Quality of libraries were determined via Agilent 4200 Tapestation using a High Sensitivity D1000 ScreenTape Assay kit (Agilent Technologies), and quantified by KAPA qPCR (KAPA Biosystems, Woburn, MA, United States). Approximately 60–80 million paired-end 150 bp sequence reads were generated for each library on the Illumina HiSeq 4000 platform. For data analysis, each sample was aligned to the GRCm38.p3 assembly of the Mus musculus reference from NCBI using version 2.6.0c of the RNA-seq aligner STAR (Dobin et al., 2013). Features were identified from the GFF file that came with the assembly from Gencode (Release M19). Feature coverage counts were calculated using featureCounts (Liao et al., 2014). Differentially expressed features were calculated using DESeq2 (Love et al., 2014) and significant differential expressions were those with an adjusted *P*-value [i.e., false discovery rate (FDR)] of <0.05.

### Gene Ontology and Pathway Enrichment Analyses

Grouping of target genes into molecular functions, biological processes, or cellular components was achieved by gene ontology (GO) analysis^1^ based on the DAVID database^2^. The Kyoto Encyclopedia of Genes and Genomes (KEGG)^3^ was used to understand high-level biological functions and utilities. Genes were also functionally grouped using Reactome^4^. Upstream factor analysis of the gene lists was carried out using the Ingenuity Pathway System (Qiagen, Redwood City, CA, United States). These analyses were each performed with a criterion FDR <0.05.

### Protein–Protein Interaction (PPI) Network Construction

To analyze the physical and/or functional interactions among the proteins encoded by identified differentially expressed genes (DEGs), DEGs were uploaded to the online program Search Tool for the Retrieval of Interacting Genes (STRING)^5^, and results with a minimum interaction score of 0.4 were collated. Clustering was done using the Markov Cluster Algorithm method with an inflation parameter of 2.

### RNA Extraction and RT-qPCR

Total RNA from liver tissues or cultured hepatocytes was extracted using a miRNeasy mini kit (Qiagen) and reverse transcribed using a miScript II RT kit (Qiagen) according to the manufacturer’s protocols. Transcripts were evaluated by qRT-PCR using an Eppendorf Mastercycler System and SYBR Green Master Mix (Eppendorf, Hauppauge, NY, United States). Primers are shown in Table 1. Each reaction was run in duplicate, and samples were normalized to GAPDH mRNA or 18S rRNA. Negative controls were a non-reverse transcriptase reaction and a non-sample reaction.

**TABLE 1:** Primers used for qRT-PCR.

**Gene ID**	**Accession no.**	**Primer**		**Length (bp)**
		**Fwd Seq (5′–3′)**	**Rev Seq (5′–3′)**	
Col3a1 (mouse)	NM_009930	GCCCACAGCCTTCTACACCT	GCCAGGGTCACCATTTCTC	110
LTBP1 (mouse)	NM_019919	TCAGAACAGCTGCCAGAAGG	GGCCACCATTCATACACGGA	125
MMP2 (mouse)	NM_008610	GCAGCTGTACAGACACTGGT	ACAGCTGTTGTAGGAGGTGC	182
Serpinh1 (mouse)	NM_009825	GCCGAGGTGAAGAAACCCC	CATCGCCTGATATAGGCTGAAG	120
Serpinhb8 (mouse)	NM_011459	GTGAAGCCAATGGGTCTTTTGC	CAGAAGAACAGGTTTCGTGACT	79
Reln (mouse)	MMU24703	TTACTCGCACCTTGCTGAAAT	CAGTTGCTGGTAGGAGTCAAAG	73
Lum (mouse)	NM_008524	CTCTTGCCTTGGCATTAGTCG	GGGGGCAGTTACATTCTGGTG	114
CCN2 (mouse)	NM_010217	CACTCTGCCAGTGGAGTTCA	AAGATGTCATTGTCCCCAGG	111
Col1a1 (mouse)	NM_007742	GCCCGAACCCCAAGGAAAAGAAGC	CTGGGAGGCCTCGGTGGACATTAG	148
αSMA (mouse)	NM_007392	GGCTCTGGGCTCTGTAAGG	CTCTTGCTCTGGGCTTCATC	148
CCNB1 (mouse)	NM_172301	AAGGTGCCTGTGTGTGAACC	GTCAGCCCCATCATCTGCG	228
ccnb2 (mouse)	NM_007630	GCCAAGAGCCATGTGACTATC	CAGAGCTGGTACTTTGGTGTTC	114
cdc25c (mouse)	NM_009860	ATGTCTACAGGACCTATCCCAC	ACCTAAAACTGGGTGCTGAAAC	67
cdk1 (mouse)	NM_007659	AGAAGGTACTTACGGTGTGGT	GAGAGATTTCCCGAATTGCAGT	128
KIF2C (mouse)	NM_134471	ATGGAGTCGCTTCACGCAC	CCACCGAAACACAGGATTTCTC	121
CYP20A1 (mouse)	NM_030013	TGCAAGGTTGTGTACCTGGAC	TGATAGGACCCTCCCCTAAAAC	104
CYP2C55 (mouse)	NM_028089	AATGATCTGGGGGTGATTTTCAG	GCGATCCTCGATGCTCCTC	111
CYP4A31 (mouse)	NM_201640	AGCAGTTCCCATCACCGCC	TGCTGGAACCATGACTGTCCGTT	276
CCL12 (mouse)	NM_011331	GTCCTCAGGTATTGGCTGGA	GGGTCAGCACAGATCTCCTT	181
CCL3 (mouse)	NM_011337	TGAAACCAGCAGCCTTTGCTC	AGGCATTCAGTTCCAGGTCAGTG	125
CCL5 (mouse)	NM_013653	AGATCTCTGCAGCTGCCCTCA	GGAGCACTTGCTGCTGGTGTAG	170
TIMP-1 (mouse)	NM_001044384	CCTATAGTGCTGGCTGTGGG	GCAAAGTGACGGCTCTGGTA	136
TREM-1 (mouse)	AF241219	CCCTAGTGAGGCCATGCTAC	GGAAGAGCACAACAGGGTCA	110
CCN2 (human)	NM_001901	AATGCTGCGAGGAGTGGGT	GGCTCTAATCATAGTTGGGTCT	115
Col1a1 (human)	NM_000088	GAACGCGTGTCATCCCTTGT	GAACGAGGTAGTCTTTCAGCAACA	91
HNF4α (human)	NM_178849	ACAGCAGCCTGCCCTCCAT	GCTCCTTCATGGACTCACACAC	143
Albumin (human)	A06977	TGCTATGCCAAAGTGTTCGATG	TTGGAGTTGACACTTGGGGT	159
CYP7A1 (human)	NM_000780	GCTACTTCTGCGAAGGCATTT	CATCATGCTTTCCGTGAGGG	131
18S (mouse, human)	X03205	GGTGAAATTCTTGGACCGGC	GACTTTGGTTTCCCGGAAGC	196
GAPDH (mouse, human)	NM_002046	TGCACCACCAACTGCTTAGC	GGCATGGACTGTGGTCATGAG	87

### Statistical Analysis

Experiments were performed at least twice in duplicate or triplicate, with data expressed as mean ± SEM. Fluorescence images were scanned and quantified using ImageJ software (NIH). Data from qRT-PCR, NTA, and imaging were analyzed by student’s *t*-test using Sigma plot 11.0 software (SPSS Inc., Chicago, IL, United States). Statistics for RNA-seq analysis are described above. *P-*values <0.05 were considered statistically significant.

## Results

### Characterization of EVs From Mouse AML12 Hepatocytes

**FIGURE 1:**
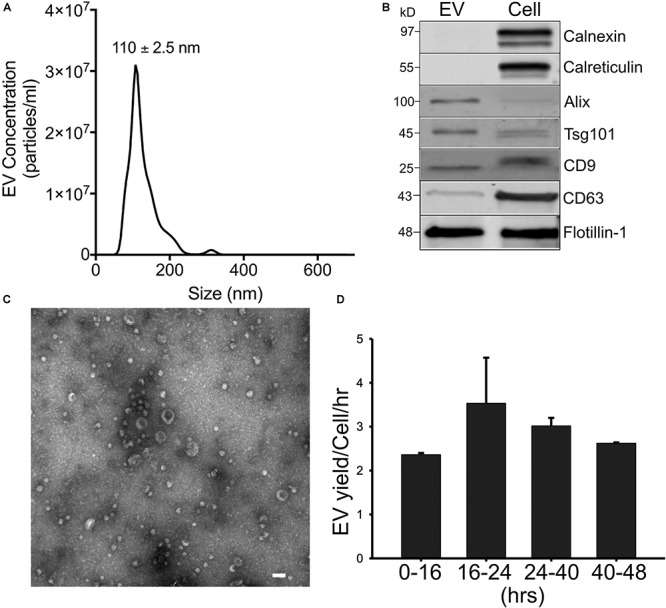
Characterization of mouse hepatocyte-derived EVs. Mouse AML12 hepatocytes were grown under serum-free conditions for 36 h after which EV^Norm^ were isolated by ultra-centrifugation of conditioned medium and characterized by **(A)** NTA [mean ± SEM for particle diameter (nm) is indicated]; **(B)** comparative Western blot with AML12 cell lysates for common EV- or cell-associated proteins (25 μg EV or cellular protein per lane; uncropped data are shown in Supplementary Figure 1); or **(C)** TEM (×56,000; scale bar = 100 nm). **(D)** EV concentration in conditioned medium from AML12 cells that were maintained under serum-free conditions for 16 h, 24 h (with a medium exchange at 16 h), 40 h (with medium exchanges at 16 and 24 h), or 48 h (with medium exchanges at 16, 24, and 40 h). The mean EV output per cell per hour over 0–16, 16–24, 24–40, or 40–48 h was computed by expressing particle number at each collection (from NTA analysis) as a function of the cell number (from cell count at each collection time) and the duration of the incubation for EV collection (8 or 16 h).

AML12 hepatocyte EVs (“EV^Norm^”) isolated from 36 to 48-h conditioned medium under serum-free conditions had a mean diameter of 110 ± 2.5 nm with a size range of approximately 50–300 nm as assessed by NTA (Figure 1A). Western blot confirmed the presence of EV-associated proteins such as CD9, CD63, Alix, flotillin, and TSG101 (Figure 1B and Supplementary Figure 1) and we have previously reported that these EVs are also positive for asialoglycoprotein receptor (Chen et al., 2018a) which is expressed exclusively in hepatocytes and has been used by others to characterize or identify hepatocyte-derived EVs (Rega-Kaun et al., 2019). Moreover, whereas calreticulin or calnexin were readily detected in AML-12 cell lysates, they were not detected in EV^Norm^ demonstrating that the EV preparations were not overtly contaminated with cytoplasmic or cytosolic components (Figure 1B). A very similar size range to that determined by NTA was obtained by TEM which also confirmed the vesicular nature of the purified nanoparticles (Figure 1C). As assessed by NTA of conditioned medium from AML12 cells that was removed and replenished at fixed time points during cell culture, there was a consistent output of EV^Norm^ when the cells were maintained in serum-free conditions for up to 48 h (Figure 1D). This demonstrated that the cell culture conditions did not compromise EV^Norm^ production and hence for the experiments described below, EV^Norm^ were collected from AML12 cells that had been maintained in serum-free conditions for up to 48 h in culture.

### Therapeutic Effects of EV^Norm^ *in vivo*

**FIGURE 2:**
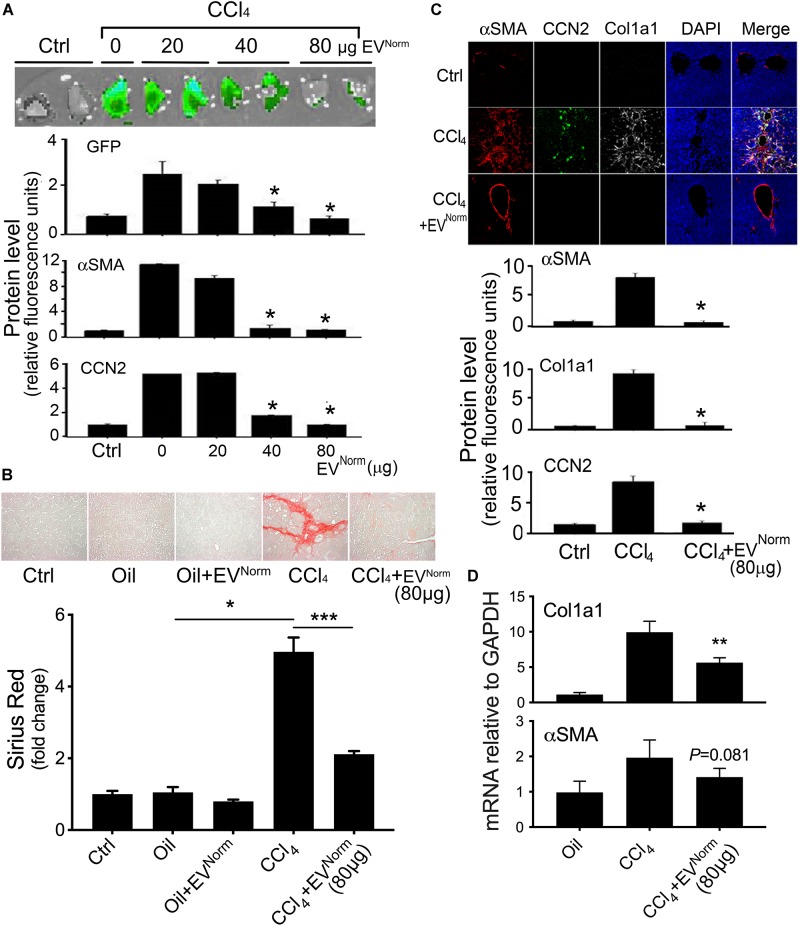
Therapeutic effects of EV^Norm^ in CCl_4_-mediated liver fibrogenesis or fibrosis. **(A)** TG CCN2-GFP mice received oil (Ctrl) or CCl_4_ over 8 days and EV^Norm^ (0–80 μg i.p. q.o.d.) on the “off days” until Day 9. Animals were sacrificed two days after the last injection. Pieces of fresh liver lobe were imaged for GFP (upper) while liver sections were stained for GFP, αSMA or CCN2 by IHC with fluorescence for each protein quantified by imaging (lower) (^∗^*P* < 0.05 vs. CCl_4_ + 0 μg EV). **(B)** Wild type Swiss Webster mice received 5 weeks of oil or CCl_4_, with or without EV^Norm^ (80 μg/25 g i.p. q.o.d.) over the last 2 weeks. The figure shows Sirius red staining of representative liver sections (upper) and quantification of the staining (lower; ^∗^*P* < 0.05; ^∗∗∗^*P* < 0.005). **(C)** Immunohistochemical detection of Col1a1, αSMA or CCN2 in liver sections (upper) and the quantified fluorescent signal assessed by scanning (lower) (^∗^*P* < 0.05 vs. CCl_4_). The residual staining for αSMA in the CCl_4_ + EV^Norm^ group reflects constitutive production of αSMA by vascular smooth muscle rather induced production in activated HSC. **(D)** qRT-PCR for Col1a1 or αSMA of total liver RNA (^∗∗^*P* < 0.01 vs. CCl_4_).

**FIGURE 3:**
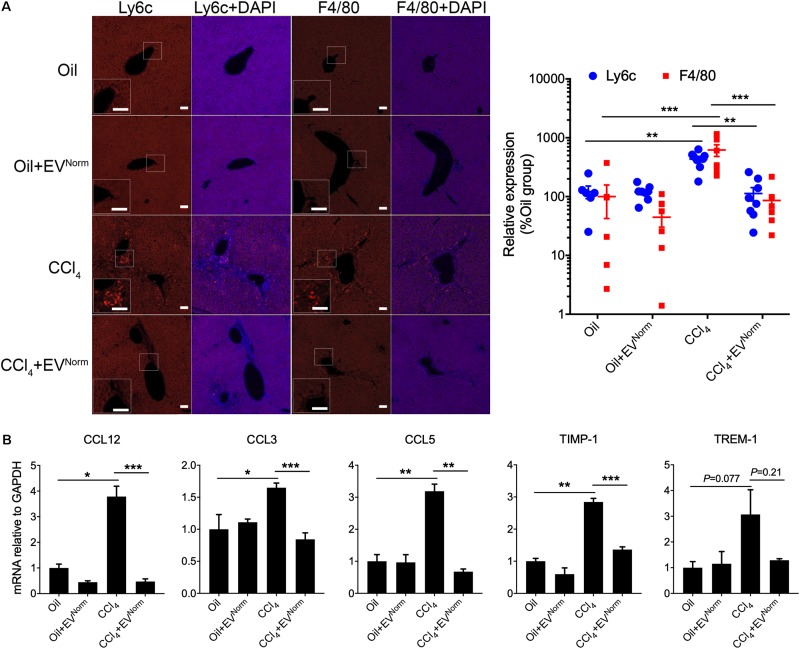
Anti-inflammatory effects of EV^Norm^ in CCl_4_-mediated liver injury. Wild type male Swiss Webster mice (*n* = 5) received oil or CCl_4_ i.p. for 9 days, with or without i.p. administration of 80 μg EV^Norm^/25 g. The figure shows **(A)** typical immunofluorescent detection of Ly6c (monocytes) or F4/80 (macrophages) (left; scale bar 50 μm), with data quantified using ImageJ software (right; each data point represents one liver section); and **(B)** mRNA expression assessed by qRT-PCR of inflammation- or fibrosis-related molecules in the liver extracts. ^∗^*P* < 0.05, ^∗∗^*P* < 0.01, ^∗∗∗^*P* < 0.005.

The effects of EV^Norm^ on fibrosing liver injury were analyzed in mice exposed to CCl_4_ which is a widely used means of inducing fibrotic responses in the rodent liver (Constandinou et al., 2005). First, as assessed by direct Xenogen imaging of GFP fluorescence in whole liver pieces, the induction of pro-fibrotic CCN2 in male TG CCN2-EGFP mice that were exposed to acute CCl_4_ injury over 8 days was dose-dependently reduced by administration of EV^Norm^ (Figure 2A). Dose-dependent suppression by EV^Norm^ on CCl_4_-induced EGFP, CCN2, or αSMA levels was also evident by immunohistochemical analysis of liver tissue sections (Figure 2A). The most effective EV^Norm^ dose (80 μg/25 g) was next tested for its effect in male wild-type Swiss Webster mice receiving CCl_4_ for 5 weeks, a chronic injury model which elevates serum levels of alanine aminotransferase and aspartate aminotransferase (Chen et al., 2018b). Hepatic fibrosis assessed by Sirius Red staining of interstitial collagen (Figure 2B) was attenuated to near-baseline levels by i.p. administration of EV^Norm^ over the last 2 weeks of the experiment and this was associated with reduced levels of CCl_4_-induced protein levels of αSMA, CCN2, or Col1a1 assessed by immunostaining (Figure 2C). CCl_4_ also caused a significant increase in hepatic Col1a1 mRNA expression that was attenuated in the presence of EV^Norm^ and a similar but non-significant trend was also seen for αSMA (Figure 2D). We further found that inflammatory responses to CCl_4_ as evidenced by increased hepatic monocyte (lyc6+) or macrophage (F4/80+) frequency was suppressed by EV^Norm^ (Figure 3A). Protein microarray analysis (not shown) indicated that EV^Norm^ dampened the CCl_4_-induced levels of tissue inhibitor of metalloprotease-1 (TIMP-1) and of the pro-inflammatory factors C-C motif chemokine ligand (CCL)3, CCL5, and CCL12, all except the latter of which have been previously shown to be associated with inflammation and fibrosis of the liver (Berres et al., 2010; Heinrichs et al., 2013; Thiele et al., 2017). Accordingly, the same targets were analyzed by RT-PCR with the result that expression of their respective CCl_4_-induced mRNAs was found to be suppressed by EV^Norm^ (Figure 3B). A similar trend but one that did not reach statistical significance occurred for triggering receptor expressed on myeloid cells 1 (TREM-1) (Figure 3B), which was recently reported to drive Kupffer cell activation, inflammation and fibrosis (Nguyen-Lefebvre et al., 2018).

### RNA-Seq Analysis of EV^Norm^-Mediated Effects on Gene Expression During CCl_4_ Liver Injury

**FIGURE 4:**
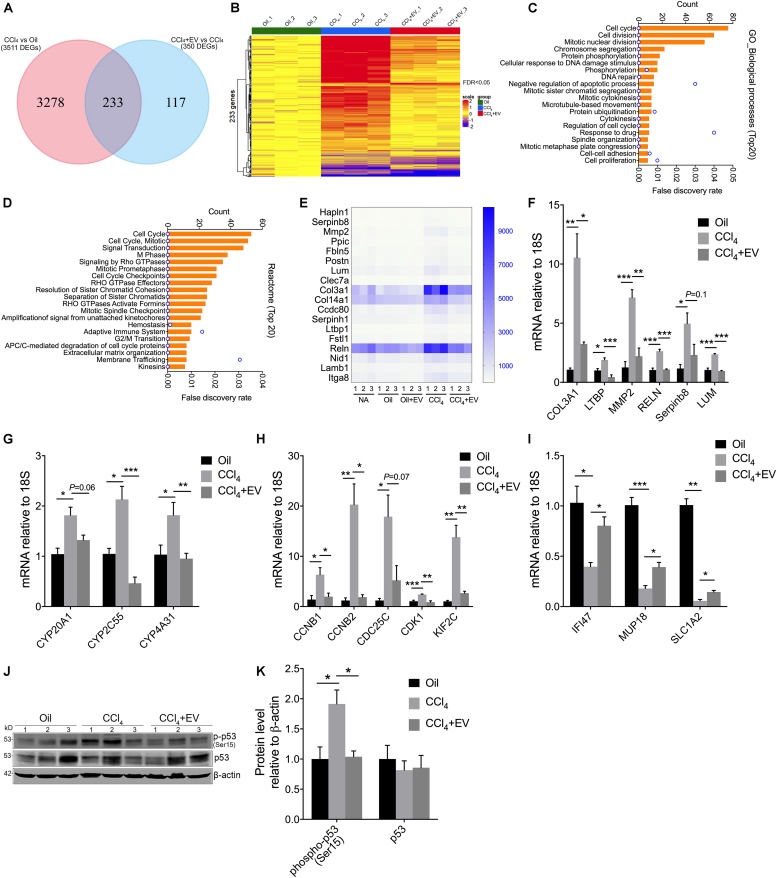
Transcriptomic changes by EV^Norm^ in long-term CCl_4_-mediated liver injury. Liver tissues from wild type Swiss Webster mice that were control or received 5 weeks of oil or CCl_4_, with or without EV^Norm^ (80 μg/25 g i.p. qod) over the last 2 weeks, were subjected to transcriptome analysis by RNA-seq as described in Methods. **(A)** Venn diagram showing DEGs between CCl_4_ vs. oil and CCl_4_ + EV^Norm^ vs. CCl_4_. **(B)** Heatmap showing pattern of expression of 233 DEGs identified in each animal of each experimental group. **(C)** Gene ontology (biological processes) analysis of the 233 DEGs showing the top 20 enriched biological processes. **(D)** Top 20 Reactome pathways of the 233 DEGs. **(E)** Heat map showing differential expression of interacting ECM-related genes identified by STRING analysis (see Supplementary Figure 2). qRT-PCR showing the effect of EV^Norm^ on either hepatic expression of CCl_4_-induced **(F)** fibrosis-related ECM genes, **(G)** cytochromes, **(H)** cell cycle-related genes, or on **(I)** CCl_4_-suppressed genes. **(J)** Liver tissue levels of total p53 or phopho-p53 detected by Western blot in light of identification of p53 by Ingenuity Pathway Analysis (Supplementary Figure 3). **(K)** Quantification of data in panel **(J)** using ImageJ. ^∗^*P* < 0.05, ^∗∗^*P* < 0.01, ^∗∗∗^*P* < 0.005.

Although our primary analysis was focused on the ability of EV^Norm^ to exert anti-fibrogenic, anti-fibrotic, and anti-inflammatory activities during liver injury *in vivo*, we next performed RNA-seq of liver tissue RNA to investigate potential broader effects of EV^Norm^ on CCl_4_-modulated hepatic gene expression. Significant (FDR < 0.05) up- or down-regulated expression of 3511 genes occurred in response to CCl_4_, 233 of which were significantly altered by EV^Norm^ (Supplementary Table 1 and Figure 4A). The differential expression of these 233 genes is represented in the heatmap shown in Figure 4B and comprised 191 genes and 42 genes that were, respectively, induced or suppressed by CCl_4_. GO (Figure 4C) and Reactome pathway (Figure 4D) analyses both indicated a significant enrichment in functions involving cell cycle or cell division, drug metabolism/detoxification, cell-cell adhesion/ECM, signal transduction, adaptive immunity, and membrane trafficking. These enrichments were consistent with the outcome of STRING analysis which revealed four major interactive clusters comprising 128 cell cycle/division-related genes, 18 ECM-related genes, 6 heat shock genes, 7 cytochrome genes, and 8 other detoxifying genes (Supplementary Figure 2). Since six genes in the ECM-related group (Figure 4E) (Col3a1, LTBP, MMP2, RELN, Serpinb8, LUM) have been reported to be involved in fibrogenesis (Preaux et al., 1999; Breitkopf et al., 2001; Krishnan et al., 2012; Alvarez Rojas et al., 2015; Carotti et al., 2017; Zhan et al., 2019), we used qRT-PCR to independently validate the ability of EV^Norm^ to dampen CCl_4_-induced expression of each of these transcripts (Figure 4F). All targets were significantly suppressed by EV^Norm^ (except Serpinb8 which nonetheless trended downward) thus confirming and extending the anti-fibrogenic properties reported above. qRT-PCR was also used to confirm the ability of EV^Norm^ to suppress CCl_4_-induced expression of cytochromes (CYP2C55, CYP4A31; Figure 4G) and of several randomly selected members of the cell cycle/cell division-related group (CCNB1, CCNB2, CDK1, KIF2C; Figure 4H), and with some targets (CYP20A1, CDC25C) showing downward but non-significant trends in expression by EV^Norm^. We also selected three CCl_4_-suppressed genes (IFI47, MUP18, SLC1A2) for qRT-PCR validation and confirmed the ability of EV^No*rm*^ to partially but significantly reverse their inhibited expression by CCl_4_. (Figure 4I). Finally, p53 – a CCl_4_-indicible tumor suppressor gene that regulates cell cycle, apoptosis, and genomic stability (Guo et al., 2013; Elgawish et al., 2015) – was ranked in first position by Ingenuity pathway analysis as a predicted upstream regulator of 40% of the EV^Norm^ DEGs (i.e., 91 of 233 genes) in CCl_4_ liver injury (Supplementary Figure 3). Consistent with this prediction, Western blot analysis of liver tissue extracts revealed that the level of CCl_4_-enhanced phospho-p53 was attenuated by EV^Norm^ (Figures 4J,K).

### Characterization and Biological Effects of EVs From Human Hepatocytes

**FIGURE 5:**
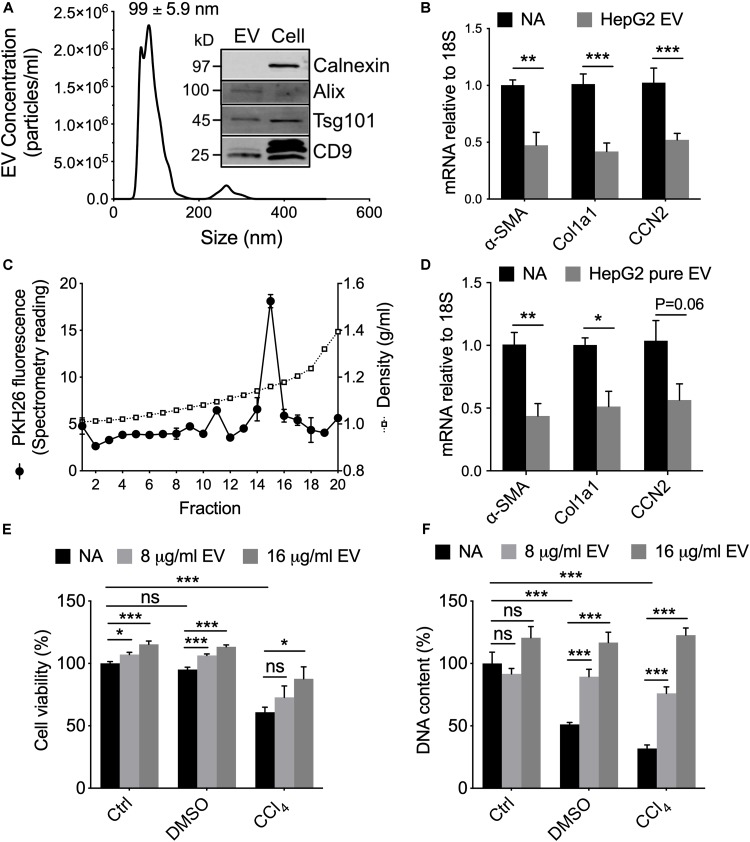
Characterization and actions of EVs from HepG2 cells. **(A)** EVs from 48-hr HepG2 cell conditioned medium (serum-free) were analyzed by NTA or Western blotting (inset, with comparison to HepG2 cell lysate; 10 μg EV or cellular protein per lane). **(B)** P3-4 mouse HSC were incubated for 42 h in the absence or presence of HepG2 cell-derived EVs (8 μg/ml) and then analyzed by RT-PCR for expression of CCN2, αSMA, or Col1a1. **(C)** PKH26-labeled EVs underwent iodixanol gradient centrifugation and **(D)** the peak fluorescent fraction was tested on mouse HSC as in panel **(B)**. **(E)** AlamarBlue viability assay or **(F)** CyQuant proliferation assay of HepG2 cells that were treated with 20 mM CCl_4_ (or 0.02% DMSO carrier) for 24 h followed by 8 or 16 μg/ml HepG2 cell-derived EVs for the following 24 h. ^∗^*P* < 0.05, ^∗∗^*P* < 0.01, ^∗∗∗^*P* < 0.005.

To substantiate that human hepatocyte EVs exert similar effects *in vitro* as their mouse counterparts, we first characterized EVs from the immortalized non-tumorigenic human HepG2 hepatocyte cell line. HepG2 cell-derived EVs purified from serum-free conditioned medium had a mean diameter of 91.2 ± 1.3 nm, with the majority of particles falling in the 50–150 nm range (Figure 5A). The EVs were positive by Western blot analysis for a variety of EV-associated proteins but the cytosol-associated protein calnexin was absent from EVs and restricted to cell lysates (Figure 5A). When incubated with activated mouse HSC *in vitro*, the HepG2 cell-derived EVs caused the transcript levels of αSMA, Col1a1 or CCN2 to be significantly suppressed (Figure 5B). To confirm that these responses were most likely attributable to the purified EVs themselves rather than a contaminant, the HepG2 cell-derived EVs were further enriched using an iodixanol gradient (Figure 5C) after which their ability to inhibit gene expression in HSC was shown to be essentially maintained (Figure 5D).

*In vivo* administration of fluorescently labeled EV^Norm^ in mice results in their localization to various hepatic target cells including HSC and hepatocytes (Chen et al., 2018a). Since we have shown that EV^Norm^ reverses ethanol/TNFα-mediated suppression of cell proliferation in hepatocytes (Chen et al., 2018a), we evaluated viability and proliferation of HepG2 cells that had been exposed *in vitro* to CCl_4_ (or DMSO carrier) in the presence or absence of HepG2 cell-derived EVs. The reduced viability caused by CCl_4_ was significantly and dose-dependently reversed by HepG2 cell-derived EVs (Figure 5E). Whereas DMSO caused a reduction in HepG2 cell proliferation, this was exacerbated even further by CCl_4_ but the deleterious effects of both agents was dose-dependently reversed by HepG2 cell-derived EVs (Figure 5F). HepG2 cell-derived EVs also promoted a slight but significant increase in the viability of HepG2 cells maintained under control non-injurious conditions (Figure 5E). Similar data were observed for CCl_4_-treated AML12 cells exposed to EV^Norm^ or HepG2 cell-derived EVs (data not shown).

**FIGURE 6:**
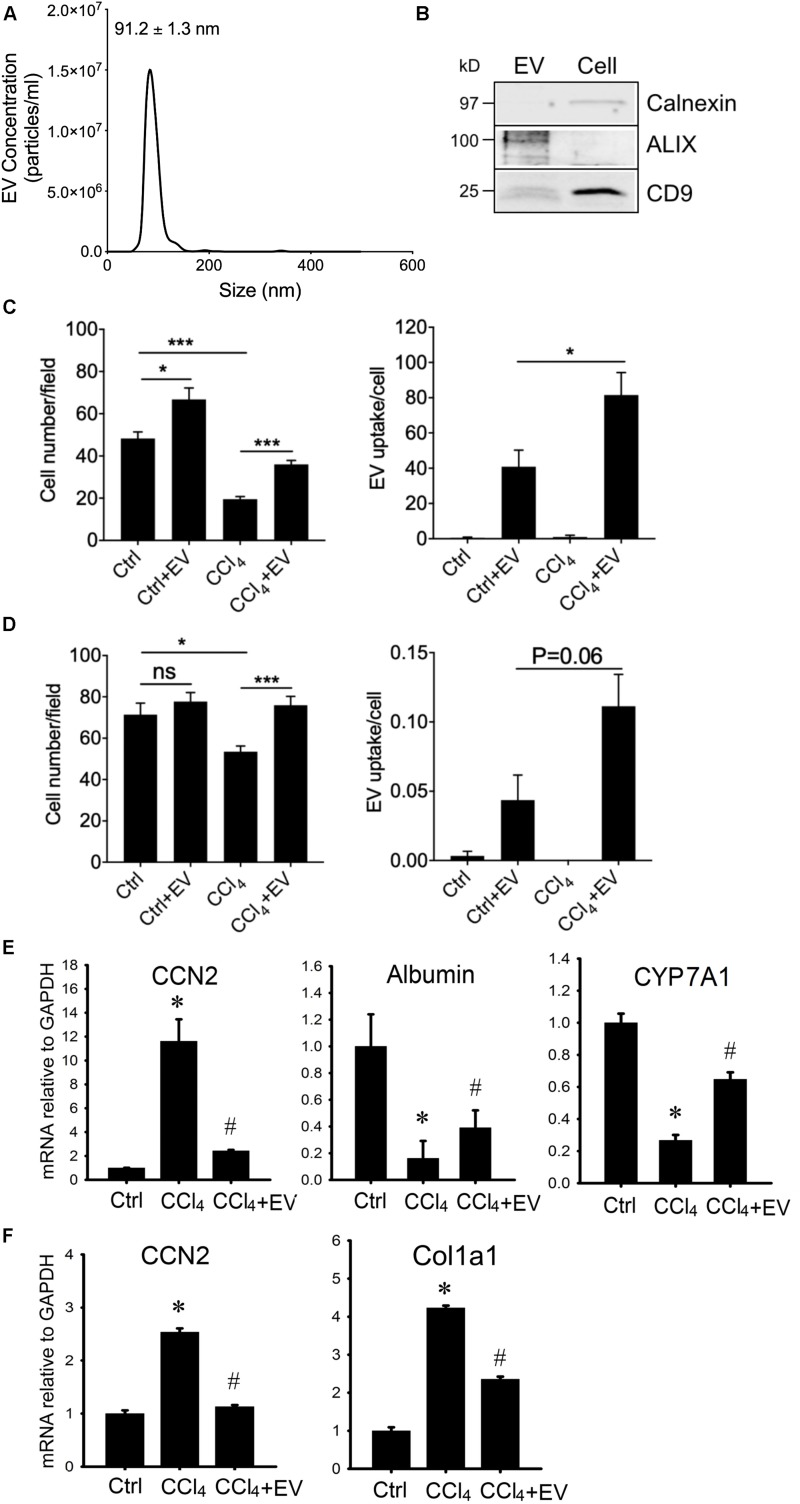
Characterization and actions of EVs from PHH. EVs present in 24-h serum-free conditioned medium from PHHs were analyzed by **(A)** NTA or **(B)** Western blotting (the latter compared to PHH lysate; 10 μg EV or cellular protein per lane; uncropped data are shown in Supplementary Figure 4). Purified PHH-derived EVs were labeled with PKH26 and incubated for 24 h with **(C)** PHHs or **(D)** LX-2 HSC that had been exposed to 20 mM CCl_4_ for the preceding 24 h. Cells were stained with DAPI and viewed using fluorescence microscopy with data quantified for total cell number (left) or the frequency of PKH26-positive cells (right). ^∗^*P* < 0.05, ^∗∗∗^*P* < 0.005. **(E)** PHHs or **(F)** LX-2 HSC were serum-starved for 12 h and then treated for 36 h with 20 mM CCl_4_ in the presence or absence of PHH-derived EVs (4 × 10^8^ particles/ml) after which mRNA was assessed by qRT-PCR. ^∗^*P* < 0.05 vs. Ctrl; #*P* < 0.05 vs. CCl_4_.

Since HepG2 cells are an immortalized cell line, we next use PHHs as an alternative source for human hepatocyte EVs. PHH-derived EVs purified from serum-free medium were 99 ± 5.9 nm in diameter (approx. range 50–150 nm) as assessed by NTA (Figure 6A) and when analyzed by Western blot they were positive for common EV proteins but not calnexin which was present exclusively in PHH lysates (Figure 6B and Supplementary Figure 4). PHH-derived EVs were then labeled with PKH26 and incubated *in vitro* with PHHs or human LX-2 HSC in the presence or absence of CCl_4_. The number of PHHs was significantly greater in the presence of EVs under both basal conditions and after exposure of the cells to CCl_4_, the latter of which otherwise caused a ∼60% decrease in overall cell number during the 48-h experiment (Figure 6C). This outcome generally resembled the effect of EV^Norm^ or HepG2 cell-derived EVs on AML12 cells or HepG2 cells (see above). The number of LX2 cells was not significantly altered by PHH-derived EVs under normal conditions but exposure of the cells to CCl_4_ resulted in a 20% decrease in cell number that was reversed in the presence of PHH-derived EVs (Figure 6D). As compared to control cultures of each cell type, there was enhanced EV binding to the cells after their exposure to CCl_4_ (Figures 6B,C), consistent with our previously reported increased binding of EV^Norm^ to (i) ethanol-treated mouse hepatocytes *in vitro*, (ii) mouse hepatocytes recovered from fibrotic livers *in vivo*, or (iii) activated mouse HSC *in vitro* or *in vivo* (Chen et al., 2018a). PHH EVs partially or completely reversed CCl_4_-mediated alterations of CCN2, albumin or CYP7A1 expression in PHHs (Figure 6E) or of CCN2 or Col1a1 expression in LX-2 HSC (Figure 6F).

Thus, EVs from human hepatocytes restored alterations in gene expression associated with injury in human hepatocytes or fibrogenesis in human or mouse HSC.

## Discussion

The emerging field of EV biology has provided a new impetus for research of liver physiology and pathology, and virtually every hepatic cell type has now been identified as an EV producer and/or recipient cell (Lemoinne et al., 2014; Hirsova et al., 2016b; Cai et al., 2017; Maji et al., 2017; Szabo and Momen-Heravi, 2017). Unfortunately, as in other organ systems, there is a dearth of information regarding the true nature of EV signaling in the liver and between the various hepatic cell types because few models have yet to be developed that would allow their various EV populations to be either tracked from the time of their initial biogenesis in donor cells to their uptake and action in recipient cells or functionally compromised *in vivo.* Despite these limitations, accumulating evidence supports a role for liver-derived EVs in regulating hepatic function. For example mouse hepatocyte exosomes promote hepatocyte proliferation *in vitro* or after acute liver injury *in vivo* caused by ischemia reperfusion (Nojima et al., 2016). Our previous (Chen et al., 2018a) and current findings for mouse or human hepatocyte EVs show that, *in vitro*, they are anti-fibrogenic for activated HSC, and reverse ethanol- or CCl_4_-suppressed proliferation or viability or alterations in gene expression in hepatocytes. Indeed, the data presented here show that hepatocyte EVs possess therapeutic properties that are quite consistent across three hepatocyte producer cell types (AML12, HepG2, PHH) from two species of origin of EV producer cell (human, mouse) and across two target cell types (HSC, hepatocytes) from two species of origin (human, mouse).

By performing a focused analysis of fibrosis-related molecular targets and cellular processes in toxic injury *in vivo* or *in vitro*, we showed that in the liver, mouse hepatocyte EVs are anti-fibrogenic, anti-fibrotic, suppress monocyte/macrophage infiltration, dampen expression of inflammatory mediators, and restore toxin-induced changes in hepatocyte function and HSC activation. The latter two properties were also elaborated by human hepatocyte EVs in *in vitro* models suggesting that the therapeutic properties are evolutionary conserved. It is currently unclear why, in CCl_4_-treated mouse liver, EV^Norm^ appears to target the αSMA protein more efficaciously than its corresponding mRNA but this may point to the targeting of αSMA post-transcriptional regulation by the EV^Norm^ molecular payload. With respect to mRNA targets, RNA-seq analysis enabled us to undertake a comprehensive analysis of liver genes that were targeted by EV^Norm^ in mice with CCl_4_-induced hepatic fibrosis. In addition to identifying a further slate of pro-fibrogenic targets, we found that EV^Norm^ action was also associated with the altered expression of genes associated with ECM, detoxification, cell cycle/cell division, signal transduction, adaptive immunity, and membrane trafficking. Thus, the transcriptomic landscape of genes targeted by EV^Norm^ was diverse, notwithstanding the fact many were associated with p53, consistent with its level of activation being a target of EV^Norm^.

Most previous studies on hepatocyte EVs have addressed their pathogenic properties when produced by cells that are stressed, injured, infected, or tumorigenic. For example, (i) exosomes are used by HCV or HEV as a mean of egress and transmission from and between hepatocytes (Tamai et al., 2012; Ramakrishnaiah et al., 2013; Nagashima et al., 2014), (ii) HCV-infected hepatocytes produce EVs that promote T follicular regulatory cell expansion (Cobb et al., 2017) and which contain miR19a which drives HSC activation via the STAT3-TGF-β pathway (Devhare et al., 2017), (iii) EVs from HBV-infected or alcohol-treated hepatocytes stimulate macrophage activation and immune function (Momen-Heravi et al., 2015; Kouwaki et al., 2016; Verma et al., 2016), (iv) exosomes derived from CCl_4_-treated hepatocytes increase toll-like receptor three expression in HSC, leading to increased IL-17A production by γδ T cells and an exacerbation of liver fibrosis (Seo et al., 2016), (v) palmitate- or lysophosphatidylcholine-treated hepatocytes produce ceramide-enriched EVs (Kakazu et al., 2016), containing C-X-C motif ligand 10 (Ibrahim et al., 2016), are released from the cell by mixed lineage kinase 3 (Ibrahim et al., 2016) and have a broad effect on target cells including stimulation of chemotaxis and production of inflammatory mediators in macrophages (Hirsova et al., 2016a; Kakazu et al., 2016), induction of migration and tube formation in endothelial cells *in vitro* and angiogenesis in mice (Povero et al., 2013), and activation of HSC via delivery of miR-192 or miR-128-3p (Povero et al., 2015; Lee et al., 2017), and (vi) exosomes from hepatocellular carcinoma cells can either deliver receptor tyrosine kinases to monocytes which show improved survival due to activation of mitogen activated protein kinases (Song et al., 2016) or elicit heat shock protein-specific NK anti-tumor responses when the donor cells are treated with anti-cancer drugs (Lv et al., 2012). In contrast, our studies show that EVs from normal hepatocytes have distinct biological properties resulting in therapeutic outcomes for liver cell damage and fibrosis. Future studies will need to provide clarity on how fibrosis and more severe forms of liver pathology are regulated by the balance between different populations of EVs from the various intact and injured hepatic cell types. It will also be interesting to determine if endogenously released EV^Norm^, along with EVs from other hepatic cells, play a more nuanced role by intrinsically limiting hepatocellular damage and pathogenesis after injury. Along these lines, the ability of hepatocyte EVs to enhance hepatocyte function (e.g., HepG2 cell viability, PHH cell number) even under basal conditions may be attributable to the collection of EVs from cells under culture conditions that are not absolutely identical to those used for subsequent biological assays, thereby manifesting a modest therapeutic benefit.

We have recently shown that serum EVs are anti-fibrogenic and anti-fibrotic in the same *in vitro* or *in vivo* models (Chen et al., 2018b). Although high fidelity animal models to monitor hepatocyte EV release and distribution are currently lacking, emerging evidence suggests that EVs harboring hepatocyte markers are detectable in the circulation, at least in certain liver diseases (Povero et al., 2014; Rega-Kaun et al., 2019). Hence in the future it will be of interest to determine if EV^Norm^ are present in the circulation and thereby contribute to the pool of therapeutic EVs in serum. We previously identified miR-34c, -151, -483, or -532 as potential therapeutic cargo molecules in serum EVs (Chen et al., 2018b) and, while detailed studies are needed in the future, our preliminary studies have implicated some of the same miRs in EV^Norm^ (Chen et al., 2016a). That said, it is likely that additional cargo molecules in EV^Norm^ contribute to the therapeutic actions of EV^Norm^ on parenchymal and non-parenchymal liver cells, as well as on the various infiltrating cell types. Indeed, as recently emphasized (Gho and Lee, 2017), a reductionist approach to addressing this question may have a somewhat limited outcome because EVs are highly heterogeneous at both the single vesicle and systems level (cargo, EV sub-types etc.). Furthermore, we documented a broad response in cellular functions and gene expression in response to EV^Norm,^ and it is likely that multiple interacting molecules in the EV molecular payload (miRs, mRNAs, proteins) are collectively involved, both directly and indirectly. For these many reasons, a holistic approach (Gho and Lee, 2017) will need to be undertaken to comprehensively determine the molecular basis for EV^Norm^ actions. Proteomic analysis of exosomes derived from rat or mouse hepatocytes previously revealed the presence of approximately 250 proteins with enrichment for functional activities including oxidoreductase, GTPase, iron-binding, lipid binding, intracellular transport, protein folding, stress response, cellular homeostasis and lipid metabolism (Conde-Vancells et al., 2008). We speculate that the combinatorial delivery by EV^Norm^ of a similar repertoire of proteins, together with EV-derived miRs and mRNAs, underlies the net therapeutic properties of EV^Norm^.

Finally, many studies have started to emerge showing that toxic liver injury, inflammation, hepatic fibrosis and HSC activation are ameliorated by EVs produced by various stem cells (SC) including umbilical cord mesenchymal SC (Li et al., 2013; Jiang et al., 2018), adipose-derived mesenchymal SC (Qu et al., 2017), embryonic SC-derived mesenchymal stromal cells (Mardpour et al., 2018), amnion-derived mesenchymal SC (Ohara et al., 2018), pluripotent SC (Povero et al., 2019), bone marrow mesenchymal SC (Rong et al., 2019), and liver SC (Bruno et al., 2019). As this research area expands, it will be important to determine if common mechanisms in these EV populations account for their shared therapeutic effects in the liver and the extent to which there is mechanistic overlap with anti-fibrogenic EVs from differentiated cells such as either hepatocytes as reported here or quiescent HSC as reported previously (Chen et al., 2014, 2015, 2016b).

In summary, hepatocyte EVs have anti-fibrotic actions *in vivo* that are associated with attenuated HSC activation and fibrogenesis, hepatocyte recovery, reduced levels of hepatic monocytes and macrophages, and attenuated expression of inflammatory mediators, as well as regulation of genes associated with ECM, detoxification, cell cycle/cell division, signal transduction, adaptive immunity, and membrane trafficking. These novel findings show that hepatocyte EVs hold promise as an innovative therapy for acute or chronic liver injury.

## Data Availability Statement

The RNA-seq datasets generated for this study can be found in the GEO accession GSE136152.

## Ethics Statement

The animal study was reviewed and approved by the Institutional Animal Care and Use Committee of Nationwide Children’s Hospital (Columbus, OH, United States).

## Author Contributions

XL performed the study design, acquisition and analysis of the data, figure preparation, and drafting and editing the manuscript. RC and SK performed the experiment planning, data acquisition, and manuscript editing. DB was responsible for the study concept and design, interpretation of the data, drafting and revising the manuscript, funding, and study supervision.

## Conflict of Interest

The authors declare that the research was conducted in the absence of any commercial or financial relationships that could be construed as a potential conflict of interest.

## Footnotes

1 http://geneontology.org/

2 https://david.ncifcrf.gov/

3 https://www.genome.jp/kegg/

4 https://reactome.org/

5 https://string-db.org/
