# Supplementary figures and images for: Extracellular Vesicles From Hepatocytes Are Therapeutic for Toxin-Mediated Fibrosis and Gene Expression in the Liver
Source: Front Cell Dev Biol. 2020 Jan 10;7:368. doi: 10.3389/fcell.2019.00368 (PMC6966099; doi:10.3389/fcell.2019.00368)

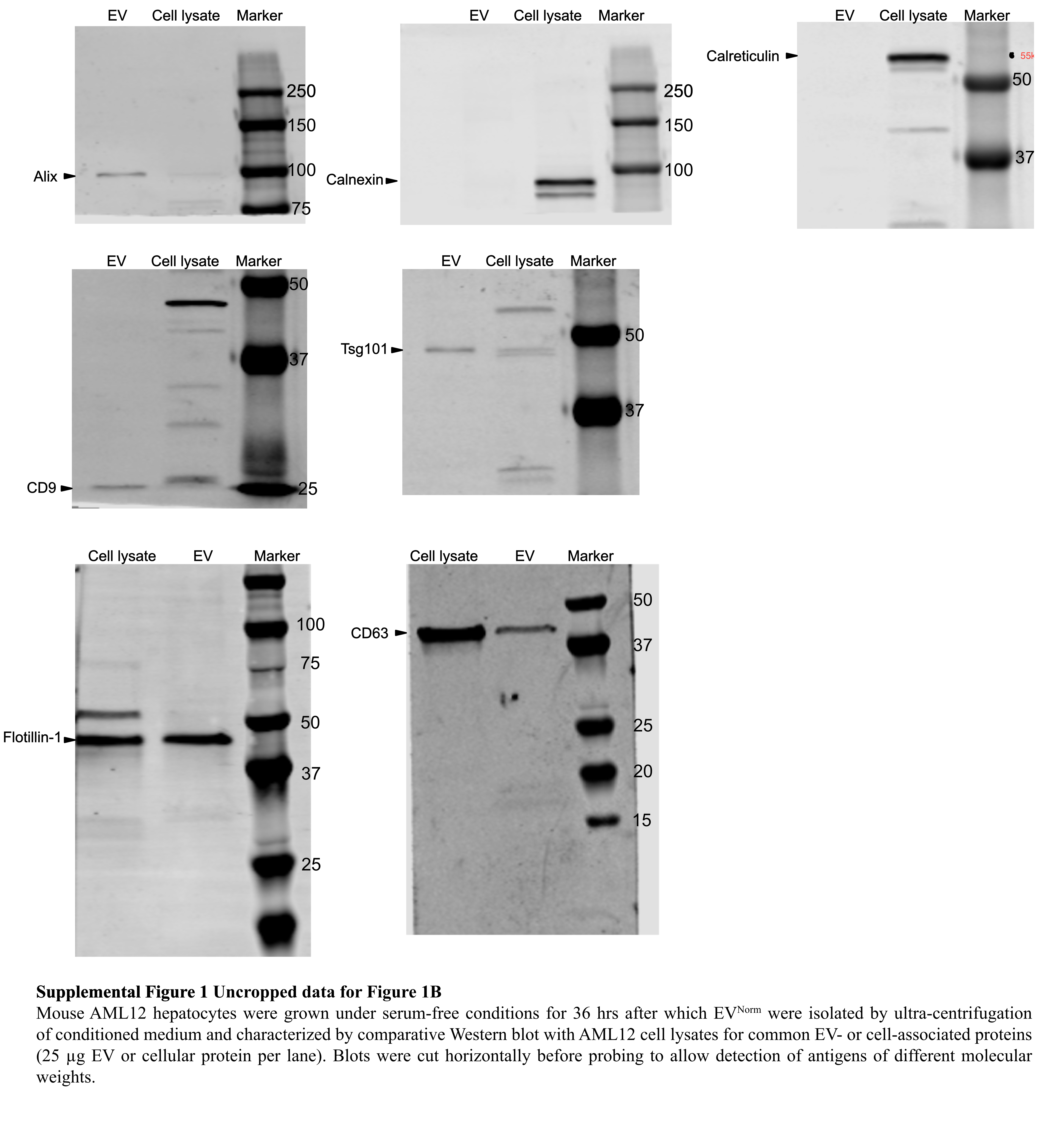

Supplement: Supplementary file 2 [file Image_1.TIFF]

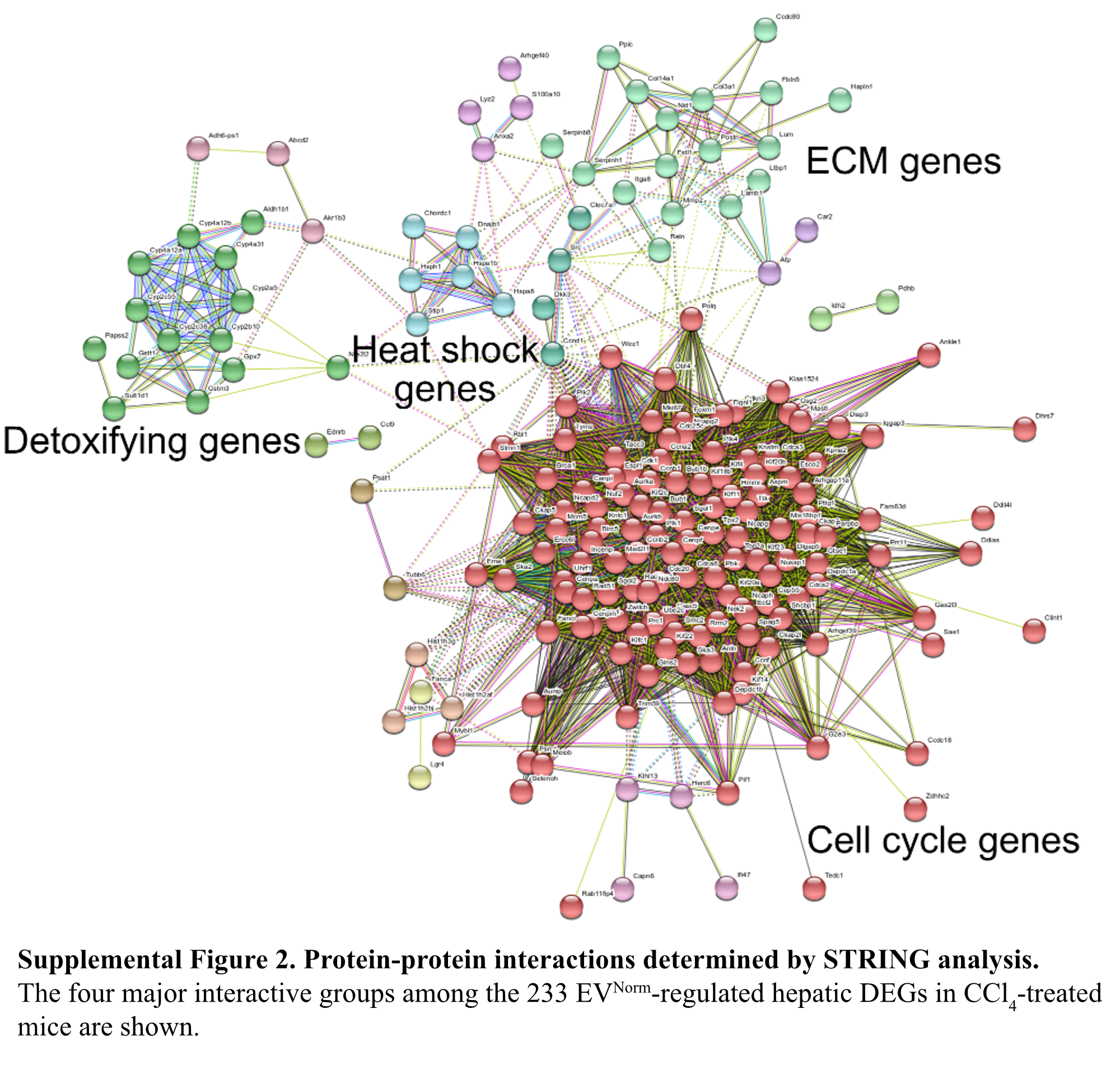

Supplement: Supplementary file 3 [file Image_2.TIFF]

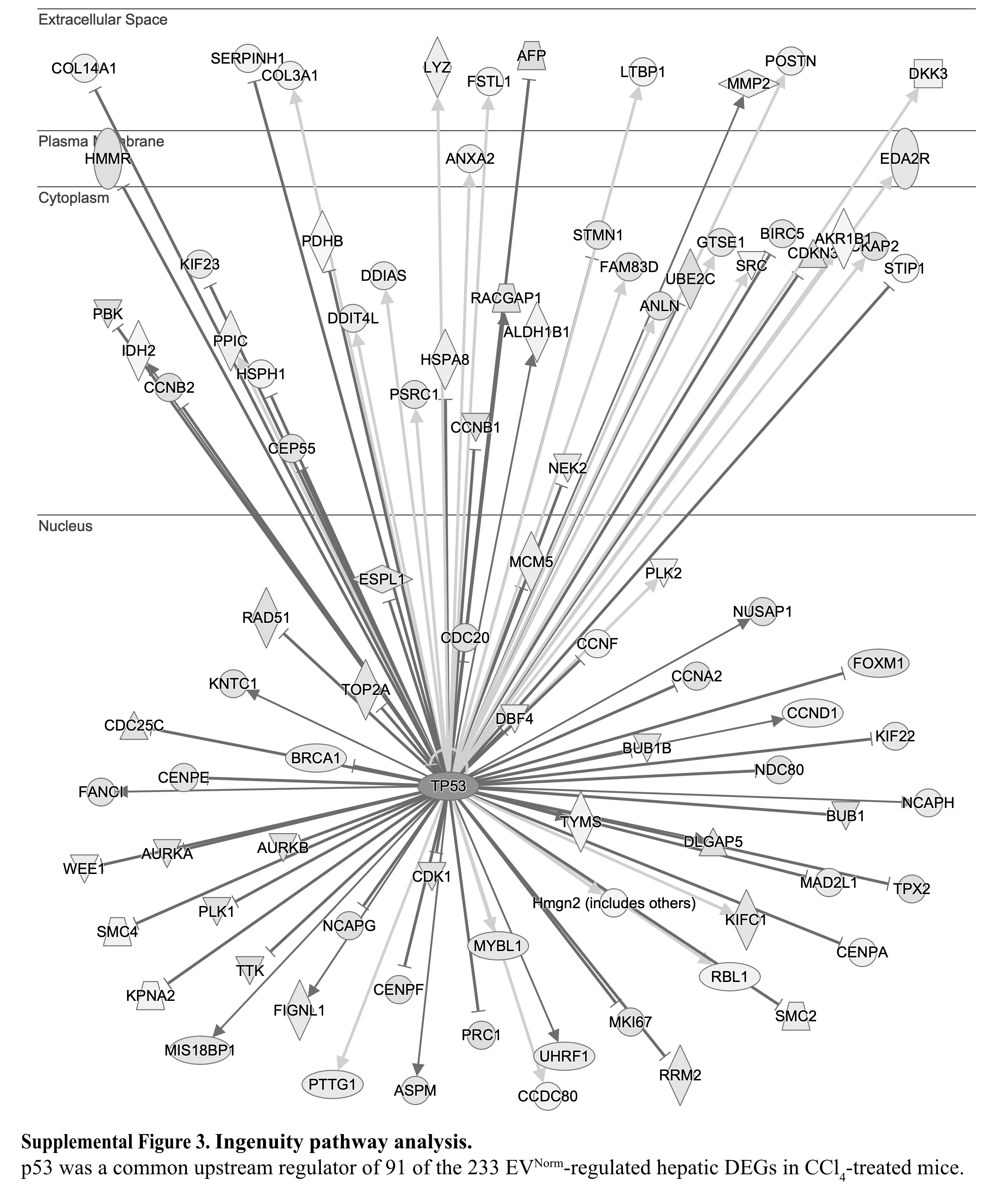

Supplement: Supplementary file 4 [file Image_3.TIFF]

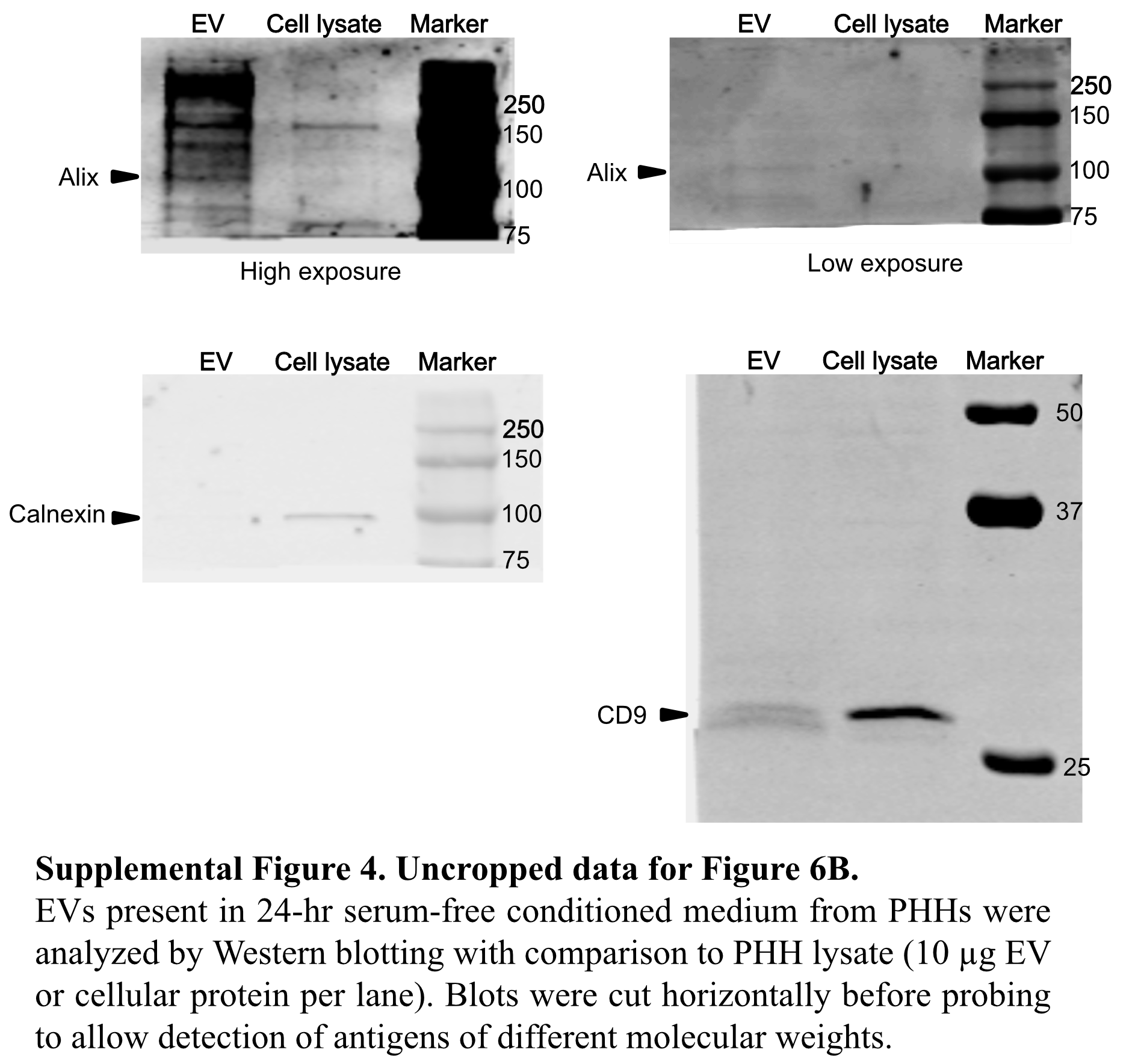

Supplement: Supplementary file 5 [file Image_4.TIFF]
